# Alcohol-induced KDM5B activation in hepatocytes drives pathogenic cell–cell communication, leading to loss of liver function

**DOI:** 10.1097/HC9.0000000000000771

**Published:** 2025-08-15

**Authors:** Kruti Nataraj, Michael Schonfeld, Samson Mah, Zhuan Li, Steven Weinman, Irina Tikhanovich

**Affiliations:** 1 Department of Internal Medicine, University of Kansas Medical Center, Kansas City, Kansas, USA; 2 The Key Laboratory of Model Animals and Stem Cell Biology in Hunan Province, Changsha, Hunan, China; 3 The Key Laboratory of Study and Discovery of Small Targeted Molecules of Hunan Province, Changsha, Hunan, China; 4 Department of Pharmacy, Hunan Normal University School of Medicine, Changsha, Hunan, China; 5 Kansas City VA Medical Center, Kansas City, Missouri, USA

**Keywords:** ALD, C/EBPβ, liver fibrosis, macrophages, sex differences

## Abstract

**Background:**

Alcohol-associated liver disease (ALD) is a major cause of alcohol-associated mortality. Previously, we identified KDM5B as a sex-specific mediator of ALD development; however, the mechanism behind KDM5B-induced pathological changes is not established.

**Methods:**

*Kdm5b* flox/flox female mice were fed a western diet and 20% alcohol in the drinking water for 8–16 weeks (WDA). To induce KO, mice received 2×10^11^ genome copies of AAV8-CMV-Cre, AAV8-TBG-Cre, or AAV8-control. To test the role of myeloid C/EBPβ, *Cebpb^fl/fl^*, or *Cebpb^fl/fl^* Lyz2-Cre mice were fed WDA for 16 weeks.

**Results:**

We found that *Kdm5b* KO prevented alcohol-induced liver fibrosis and liver inflammation in female mice. These changes were in part mediated by hepatocyte-to-non-parenchymal cell communication changes. KDM5B in hepatocytes promoted pro-inflammatory and pro-fibrotic changes in liver macrophages, endothelial cells, and stellate cells. Moreover, KDM5B promoted alcohol-induced early increase in EpCAM-positive liver progenitors and loss of liver function at later time points of alcohol feeding. We found that loss of liver function was dependent on a hepatocyte-to-macrophage communication feedback loop. KDM5B in hepatocytes inhibited macrophage C/EBPβ expression, which in turn resulted in loss of the mature KCs phenotype and prevented the ability of KCs to support hepatocyte differentiation, ultimately leading to loss of liver synthetic function.

**Conclusions:**

KDM5B activation in hepatocytes drives pathogenic cell–cell communication, leading to alcohol-induced loss of liver function in ALD.

## INTRODUCTION

Alcohol-associated liver disease (ALD) encompasses a spectrum of disorders, ranging from steatosis to steatohepatitis, fibrosis, cirrhosis, and HCC.^1^,^2^,^3^,^4^ There is still limited therapy for any stage of ALD. Recently, we have identified a unique female-specific mechanism of ALD development that involves alcohol-induced activation of demethylases KDM5B and KDM5C.^5^ However, the precise mechanism of KDM5-dependent ALD development in females is not yet fully elucidated.

KDM5B is an enzyme that demethylates lysine 4 of histone H3 at active gene promoters, resulting in transcriptional repression.^6^,^7^,^8^,^9^ We and others have identified the roles of KDM5B in fibroblast activation and fibrosis progression in liver and cardiac fibrosis.^5^,^10^,^11^ On the other hand, multiple studies suggest that KDM5B deficiency or inhibition can reduce the production of pro-inflammatory cytokines and full activation of NF-κB signaling.^12^ Thus, KDM5B can affect multiple aspects of ALD pathology.

C/EBPβ is a liver-enriched transcription factor.^13^,^14^,^15^,^16^,^17^,^18^ C/EBPβ is activated by a variety of signals that play a role in the regulation of multiple inflammatory pathways.^13^,^14^,^15^,^19^,^20^,^21^ The C/EBPβ gene is strongly upregulated by lipopolysaccharide and cytokines. Targeted inactivation of the C/EBPβ gene in the mouse results in macrophage dysfunction, likely due to loss of C/EBPβ-mediated expression of macrophage colony-stimulating factor receptor.^22^

C/EBPβ is involved in mediating inflammatory responses.^23^,^24^ C/EBPβ thus has an important role in the pathological development of various inflammation-related diseases.^23^ Interestingly, though in vitro data suggested that C/EBPβ regulates the expression of pro-inflammatory cytokines,^25^,^26^ cytokine gene expression was unchanged in macrophages from C/EBPβ KO (KO) mice after lipopolysaccharide stimulation. In contrast, anti-inflammatory gene expression depends on C/EBPβ both in vitro and in vivo.^27^ A CREB-C/EBPβ cascade induces M2-like macrophage-specific gene expression^28^ and promotes muscle injury repair.^29^ We showed previously that C/EBPβ promotes PD-L1 and other immune checkpoint gene expression in tumor-associated macrophages in vivo.^30^

In this work, we found that KDM5B in hepatocytes promotes alcohol-induced inflammation and fibrosis in females. It does so by promoting hepatocyte progenitor accumulation [ductular reaction (DR)], monocyte-derived macrophage infiltration, and by inhibiting macrophage C/EBPβ expression. Loss of macrophage C/EBPβ in turn results in loss of the mature KC phenotype and reduces the KC ability to support hepatocyte differentiation, ultimately leading to loss of liver synthetic function.

## METHODS

*Kdm5b*-floxed mice (B6/J^GptKdm5cem1C^ flox/wt) were obtained from GemPharmatech Co., Ltd, and bred to make homozygous flox/flox breeders. Mice were maintained on the B6/J^Gpt^ background.

*Cebpb*-floxed mice (BALB/cJ-*Cebpb^tm1.1Elgaz^*) were obtained from Jackson lab and backcrossed for 7 generations to the C57BL6/J background. These mice were next crossed with Lyz2-Cre mice [Lyz2-Cre (Jackson Labs, Stock No. 004781)] to generate mice lacking C/EBPβ in myeloid cells. For experiments, *Cebpb^fl/fl^* Cre/wt mice were used together with *Cebpb^fl/fl^* wt/wt littermates as a control.

All mice were housed in a temperature-controlled, specific pathogen-free environment with 12-hour light–dark cycles. All animal handling procedures were approved by the Institutional Animal Care and Use Committee at the University of Kansas Medical Center (Kansas City, KS).

For fibrosis induction, mice were treated with 200 mg/L of thioacetamide (TAA) in the drinking water for 2 months.

### WDA model

For the previously described western diet alcohol model,^31^ both male and female mice were fed ad libitum western diet (Research Diets, Inc., Cat #D12079B), and alcohol was given ad libitum in water. Mice received progressively increasing amounts of alcohol in water (3%, 10%, 15%, and 20% for 3 days each). After reaching 20%, mice continued for 8–18 weeks as indicated. Alcohol containing water was changed twice weekly. Mice on this diet consume 18–20 g/kg/day of alcohol, which corresponds to 22%–24% of total calories from alcohol; 33%–34% of total calories are coming from fat.

### Vectors

AAV-TBG-control and AAV-TBG-iCre were from VectorBiolabs and were used at 2×10^11^ genome copies per mouse (Cre/control). AAV8-CMV-Cre and AAV8-CMV-EGFP were from Vector Builder and were used at 2×10^11^ genome copies per mouse each.

### Antibodies

Anti-COL1A1 antibodies [COL1A1 (E8I9Z) Rabbit mAb #91144], anti-F4/80 [F4/80 (D2S9R) XP Rabbit mAb #70076], and anti-C/EBPβ LAP [C/EBPβ (LAP) Antibody #3087] were from Cell Signaling Technology. Anti-C/EBPβ antibodies were from SantaCruz [C/EBP beta Antibody (H-7): sc-7962]. Anti-EpCAM antibodies (ab71916) were from Abcam. HNF4 alpha/NR2A1 antibodies (NBP1-89679) were from Novus.

### Analysis of blood samples

Whole blood was collected from the retroorbital vein of mice. Serum was used to measure ALT (Pointe Scientific ALT Liquid Reagents, A7526150, Pointe Scientific) and AST (Pointe Scientific ALT Liquid Reagents, A7561450, Pointe Scientific).

### Cell isolation

Liver cells were isolated by a modification of the method described by Troutman et al.^32^ Mouse livers were digested by retrograde perfusion with Liberase TM via the inferior vena cava. The dissociated cell mixture was placed into a 50 mL conical tube and centrifuged twice at 50*g* for 2 minutes to pellet hepatocytes. The NPC-containing cell supernatant was further used to isolate KC, LSEC, and HSC in an OptiPrep gradient. KCs and endothelial cells were further purified with F4/80+ and CD146+MicroBeads (Miltenyi Biotec), respectively, according to the manufacturer’s instructions.

### Isolation of mouse peritoneal macrophages

Primary peritoneal macrophages were isolated as described previously.^33^ Mice aged 8–10 weeks were killed by CO_2_ asphyxiation. Briefly, 10 mL of sterile PBS was injected into the caudal half of the peritoneal cavity using a 25-gauge needle (beveled side up), followed by gently shaking the entire body for 10 seconds. Saline containing resident peritoneal cells was collected, and cells were plated on uncoated tissue culture plates and incubated for 60 minutes at 37 °C. Nonadherent cells were removed by washing 5 times with warm PBS. Macrophages were maintained in RPMI medium (Invitrogen) containing 10% fetal bovine serum.

### Transwell co-culture

For co-culture experiments, freshly isolated hepatocytes were seeded in 24-well Transwell (Corning Incorporated, 0.4 µm pore size) at a seeding density of 1 × 10^5^/well. Hepatocytes from *Kdm5b*-floxed mice were placed in cell inserts. Cells were transfected with a Cre recombinase-expressing vector or an empty vector control. Freshly isolated liver macrophages/LSECs/HSCs were seeded in the bottom well. The cells were then cultured for 24 hours, and the cells were harvested for RNA isolation.

### Immunohistochemistry

Liver tissue sections (5 μm thick) were prepared from formalin-fixed, paraffin-embedded samples. Immunostaining on formalin-fixed sections was performed by deparaffinization and rehydration, followed by antigen retrieval achieved by heating in a pressure cooker (121 °C) for 5 minutes in 10 mM sodium citrate, pH 6.0, as previously described.^34^ Peroxidase activity was blocked by incubation in 3% hydrogen peroxide for 10 minutes. Sections were rinsed 3 times in PBS/PBS-T (0.1% Tween-20) and incubated in Dako Protein Block (Dako) at room temperature for 1 hour. After removal of the blocking solution, slides were placed into a humidified chamber and incubated overnight with a primary antibody diluted 1:300 in Dako Protein Block at 4 °C. The antigen was detected using the SignalStain Boost IHC detection reagent (catalog #8114; Cell Signaling Technology), developed with diaminobenzidene (Dako), counterstained with hematoxylin (Sigma-Aldrich), and mounted.

### RT-PCR

RNA was extracted from livers using the RNeasy Mini Kit (Qiagen). cDNA was generated using the RNA reverse transcription kit (Applied Biosystems, Cat. #4368814). Quantitative real-time RT-PCR was performed in a CFX96 Real Time system (Bio-Rad) using specific sense and antisense primers combined with iQ SYBR Green Supermix (Bio-Rad) for 40 amplification cycles: 5 seconds at 95 °C, 10 seconds at 57 °C, 30 seconds at 72 °C. mRNA concentrations were calculated relative to *Actb*.

### Primers

**Table:** 

mActb	ATGTCACGCACGATTTCCCT	CGGGACCTGACAGACTACCT
mTnf	CTGAGACATAGGCACCGCC	CAGAAAGCATGATCCGCGAC
mCol1a1	TGGCCAAGAAGACATCCCTG	GGGTTTCCACGTCTCACCAT
mIL1b	ACGGGAAAGACACAGGTAGC	AGCTTCAGGCAGGCAGTATC
mTimp1	GTAAGGCCTGTAGCTGTGCC	AGCCCTTATGACCAGGTCCG
mTgfb1	TACGTCAGACATTCGGGAAGC	TTTAATCTCTGCAAGCGCAGC
mAlb	CTGCACACTTCCAGAGAAGGA	CAGTCTTCAGTTGCTCCGCT
mCcl2	ACCTGGATCGGAACCAAATGAG	GCTGAAGACCTTAGGGCAGAT
mCcl5	GGATTACTGAGTGGCATCCCC	TCTGACCCTGTATAGCTTCCCT
mTrem2	CTACTTTTGCTTCAGAGGCCG	CTTCCCCACTCAACACAGATG
mVsig4	CAGGTGTTTTTAGGGTGGGGT	TCATCAGGCTTGCTGTTCCTG
mEpcam	GACGACGTGGACATAGCTGA	GCTCTCCGTTCACTCTCAGG
mCd163	GCTGAGGATGTCGGTGTGAT	TCCTGAACATCTGGACACTCC
mKrt7	CTTCCCCGAATCTTTGAGGCT	ACCACATCCTGCATGTTCCG
mGpnmb	GTCCTGATCTCCATCGGCTG	TGGCTTGTACGCCTTGTGTT
mHnf4a	AGCAATGGACAGATGTGTGAGT	TTCAGATCCCGAGCCACTTG
mCebpb	TCACTTAAAGATGTTCCTGCGG	TGCTCGAAACGGAAAAGGTTC
mStab2	CCAGCTGGGTAAATGCAACA	ATATGACGGCTGGTGTCCTC
mLyve1	TTTGTTGCAAGTGGAGCAGC	GTAGCAAACAGCCAGCACAG
mIcam1	GGTGAGGTCCTTGCCTACTTG	TCACCGTGTATTCGTTTCCG
mVcam1	GGGGGCCACTGAATTGAATCT	GGAAGCTGGAACGAAGTATCC

### Cytokine array

Proteome Profiler Mouse Cytokine Array Kit (R&D Systems) was used according to the manufacturer’s instructions.

### Hydroxyproline assay

Hydroxyproline assays were performed using a hydroxyproline assay kit (Cell Biolabs Cat #STA-675) according to the manufacturer’s instructions.

### Statistics

Data were plotted and analyzed in Prism GraphPad. Comparison between different datasets was made using an unpaired 2-tailed *t* test with Welch correction and 1-way ANOVA with Tukey post hoc test. *p*<0.05 was considered to be statistically different.

## RESULTS

### Hepatocyte-specific KO of KDM5B in female mice protects from alcohol and high-fat diet-induced pathology

**FIGURE 1:**
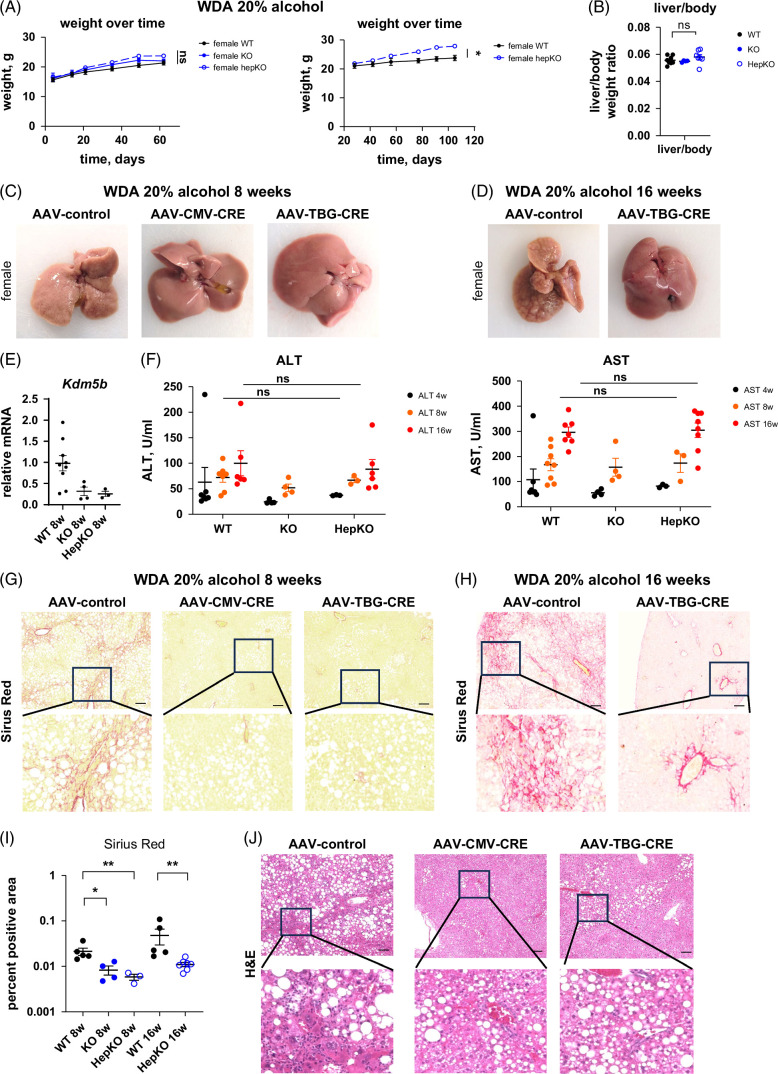
Hepatocyte KDM5B promotes western diet and alcohol induced liver pathology in female mice. *Kdm5b* fl/fl female mice were fed a high-fat western diet with 20% alcohol in the drinking water (WDA) for 8 or 16 weeks. Mice were treated with AAV8-CMV-Cre (KO), AAV8-TBG-Cre (HepKO), or AAV8-control (WT) at the beginning of alcohol feeding. (A) Weight change in these mice. N=3–8 mice per group; *p<0.05. (B) Liver/body weight ratio at the end of the feeding. (C, D) Gross liver appearance after 8 weeks (C) or 16 weeks (D) of feeding. (E) *Kdm5b* gene expression in the whole liver mRNA after 8 weeks of alcohol feeding. (F) Serum ALT and AST at indicated times. N=3–8 mice per group. (G, H) Representative images of Sirius Red staining in these mice. (I) Sirius Red positive area in these mice. N=3–8 mice per group. *p<0.05 and **p<0.01. (J) H&E staining in 3 groups of mice after 8 weeks of WDA feeding. Scale bar 100 µm. Abbreviations: AAV, adeno-associated virus; H&E, hematoxylin and eosin; KO, knockout; WT, wild type.

Previously, we observed that liver-directed, but non-cell-type-specific, knockdown of *Kdm5b* using an shRNA vector reduced alcohol-induced fibrosis development in female mice.^5^ To assess the relative contribution of hepatocytes to the phenotype, we used *Kdm5b*-floxed mice and treated them with AAV.CMV.Cre to recapitulate unselective *Kdm5b* loss and AAV.TBG.Cre to induce hepatocyte-specific KO of *Kdm5b* (Figure 1) as previously described.^35^ Mice were then fed a high-fat diet [western diet (WD)] with 20% alcohol in the drinking water (WDA model^31^) for 8 or 16 weeks. We observed that although CMV-Cre-mediated KO did not affect weight gain in these mice, hepatocyte-specific KO resulted in a small increase in weight gain (Figure 1A) with the difference becoming apparent from 8 weeks onward. Despite weight differences, all 3 groups of mice had similar liver-to-body weight ratios at the end of the experiment (Figure 1B). On gross examination, livers from control mice showed nodular appearance of the liver at 8 and 16 weeks of feeding (Figures 1C, D). In contrast, KO mouse livers appeared healthy at both time points (Figures 1C, D). We confirmed that AAV-Cre vectors resulted in significant downregulation of liver *Kdm5b* mRNA levels (Figure 1E). To assess liver injury, we measured serum ALT and AST levels in these mice (Figure 1F). We found that wild-type (WT) and KO mice had similar ALT and AST at 4, 8, and 16 weeks of feeding, suggesting that *Kdm5b* loss does not affect liver injury markers in these mice.

To assess the effect of *Kdm5b* loss on liver fibrosis, we evaluated fibrosis development by Sirius Red staining (Figures 1G, H). We found that WT mice developed pericellular liver fibrosis by 8 weeks of feeding, which was further increased at 16 weeks (Figures 1G, I). CMV-Cre-mediated *Kdm5b* KO prevented fibrosis development in these mice, in agreement with previously published studies. We found that hepatocyte-specific *Kdm5b* KO was as effective as CMV-Cre KO in preventing fibrosis development in alcohol-fed mice after 8 weeks of feeding (Figures 1G, I), and hepatocyte-specific *Kdm5b* KO greatly reduced liver fibrosis development after 16 weeks as well (Figures 1H, I).

We further assessed liver pathology by hematoxylin and eosin staining (Figure 1J). We found that at 8 weeks of feeding, WT mice developed liver steatosis and inflammation, while KO mice were protected from inflammation and showed less severe liver steatosis (Figure 1J). We found no significant difference between CMV-Cre and TBG-Cre KO mice. Taken together, these data suggest that *Kdm5b* KO in hepatocytes protects mice from inflammation and fibrosis.

### Hepatocyte-specific KO of KDM5B in female mice protects from alcohol and high-fat diet-induced liver fibrosis

**FIGURE 2:**
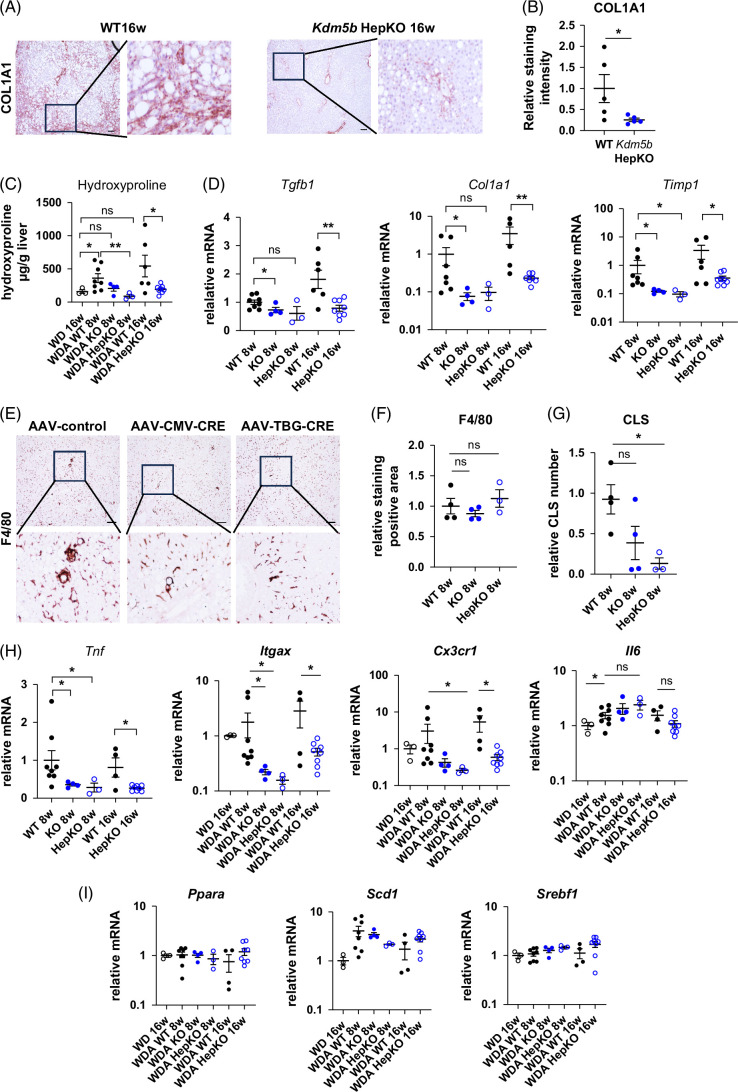
Hepatocyte KDM5B promotes alcohol-induced liver fibrosis and inflammation in female mice. *Kdm5b* fl/fl female mice were fed a high-fat western diet with 20% alcohol in the drinking water (WDA) for 8 or 16 weeks. Mice were treated with AAV8-CMV-Cre (KO), AAV8-TBG-Cre (HepKO), or AAV8-control (WT) at the beginning of alcohol feeding. (A) Representative images of Collagen 1A1 staining in WT and HepKO mice after 16 weeks of WDA feeding. (B) COL1A1-positive staining intensity in these mice. N=5 mice per group; *p<0.05. (C) Hydroxyproline levels in liver tissue. N=3–8 mice per group; *p<0.05 and **p<0.01. (D) Relative whole liver mRNA in these mice. N=3–8 mice per group; *p<0.05 and **p<0.01. (E) Representative images of F4/80 staining in WT and KO mice after 8 weeks of WDA feeding. (F) F4/80-positive staining area relative to WT controls in these mice. N=3–4 mice per group. Right: F4/80-positive staining area relative to WT controls in these mice. N=3–4 mice per group. (G) CLSs per high-power field. N=3–4 mice per group; *p<0.05. (H, I) Relative whole liver mRNA in these mice. N=3–8 mice per group; *p<0.05. Scale bar 100 µm. Abbreviations: AAV, adeno-associated virus; CLSs, crown-like structures; KO, knockout; WD, western diet; WT, wild type.

We further examined liver fibrosis and inflammation in these mice. We found that KO mice showed reduced COL1A1 protein staining assessed by immunohistochemistry (Figures 2A, B) and reduced liver hydroxyproline levels in KO ice compared to WT controls (Figure 2C). These data agree with reduced fibrosis-associated gene expression (*Tgfb1*, *Col1a1*, *T* *imp1*) in KO mice compared to WT controls at 8 and 16 weeks of feeding (Figure 2D). Collectively, this suggests that hepatocellular KDM5B greatly contributes to alcohol-induced liver fibrosis development in female mice.

Reduced fibrosis correlated with reduced markers of liver inflammation. We observed that although F4/80 staining was similar in intensity between the genotypes (Figure 2E, F), the number of crown-like structures was greatly reduced in KO mice (Figure 2G). These data correlated with reduced expression of pro-inflammatory cytokines such as TNFα, and reduced inflammation-associated gene expression (*Itgax, Cx3cr1*), but not *Il6* (Figure 2H). In contrast, *Kdm5b* KO did not greatly affect genes related to lipid metabolism, such as genes related to FA synthesis or β-oxidation (Figure 2I). Taken together, these data suggest that hepatocyte KDM5B contributes greatly to alcohol-induced inflammation and fibrosis in female mice.

### Hepatocyte KDM5B promotes pathogenic cell–cell communication in the liver

**FIGURE 3:**
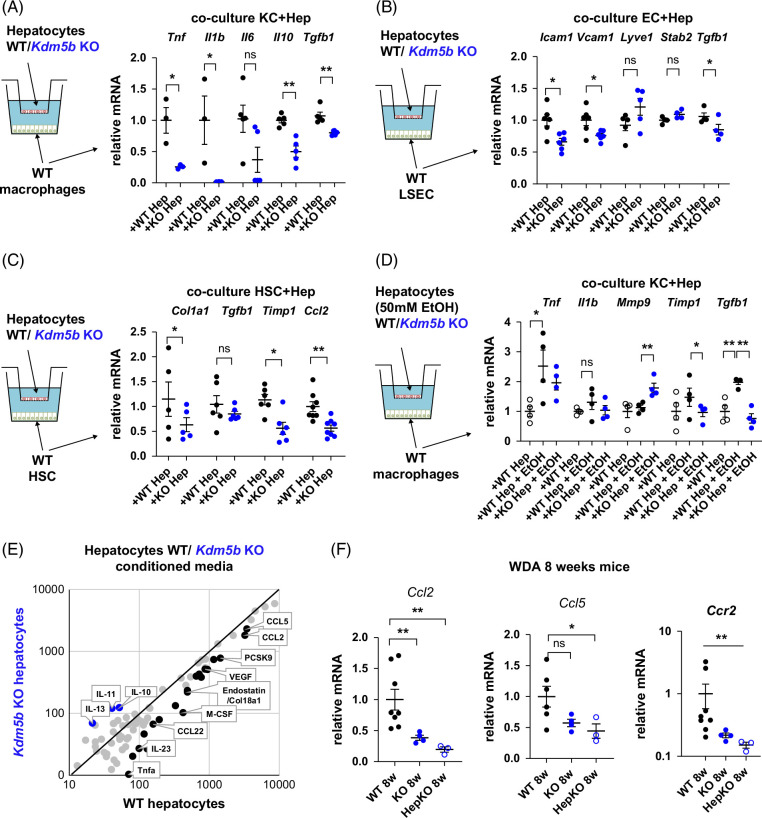
Hepatocyte KDM5B promotes fibrosis and inflammation-related gene expression changes in non-parenchymal cells. (A–D) *Kdm5b* WT or KO hepatocytes were used in a co-culture system with liver macrophages (A), liver sinusoidal endothelial cells (B), HSC (C), or liver macrophages in the presence of 50 mM alcohol as indicated (D). Relative gene expression in NPCs after 24 hours of co-culture. N=3–6 independent experiments; *p<0.05 and **p<0.01. (E) *Kdm5b* WT or KO hepatocytes' conditioned media were analyzed using a cytokine array. (F) Relative whole liver mRNA in WT and KO mice after 8 weeks of WDA feeding. N=3–8 mice per group; *p<0.05 and **p<0.01. Abbreviations: KO, knockout; NPCs, non-parenchymal cells; WDA, western diet with alcohol; WT, wild type.

To evaluate the mechanism of hepatocyte KDM5B-mediated inflammation and fibrosis development, we examined hepatocyte-to-non-parenchymal cell crosstalk using a co-culture system. We showed that compared to WT control hepatocytes *Kdm5b* KO hepatocytes reduced pro-inflammatory and pro-fibrotic gene expression in liver macrophages (F4/80-positive cells, Figure 3A), reduced adhesion molecule gene expression in sinusoidal endothelial cells (CD146-positive cells, Figure 3B) and reduced markers of HSC activation (Figure 3C) after 24 hours of co-culture using freshly isolated cells. Taken together, these data suggest that hepatocyte KDM5B may promote inflammation and fibrosis by affecting multiple non-parenchymal cells in the liver. To assess if these mechanisms exist in the presence of alcohol, we examined hepatocyte-to-macrophage crosstalk in the presence of 50 mM ethanol (Figure 3D). We found that in the presence of alcohol, macrophages displayed pro-inflammatory and pro-fibrotic gene expression changes. Moreover, compared to WT control hepatocytes, *Kdm5b* KO hepatocytes reduced pro-inflammatory and pro-fibrotic gene expression in liver macrophages, abolishing the effect of alcohol (Figure 3D).

To assess the hepatocyte secretome changes induced by *Kdm5b* KO, we performed cytokine arrays of conditioned media from freshly isolated WT and KO primary hepatocytes after 24 hours of incubation (Figure 3E). Among the top differentially regulated secreted proteins, we found that KDM5B promoted the production of CCL-2 and CCL-5, well-known to promote liver inflammation, fibrosis, and ALD development, as well as PCSK9, reported to promote metabolic liver disease. We confirmed that in mice, after 8 weeks of alcohol feeding, *Kdm5b* KO significantly downregulated *Ccl2* and *Ccl5* gene expression (Figure 3F), suggesting that hepatocyte KDM5B promotes ALD in part via upregulated *Ccl2* and *Ccl5* gene expression in hepatocytes. Moreover, CCL-2 receptor CCR2 was similarly downregulated, suggesting that *Kdm5b* KO prevented monocyte-derived macrophage infiltration into the liver, thus reducing liver inflammation (Figure 3F).

### Hepatocyte KDM5B loss results in sex-specific transcriptional changes in the liver

**FIGURE 4:**
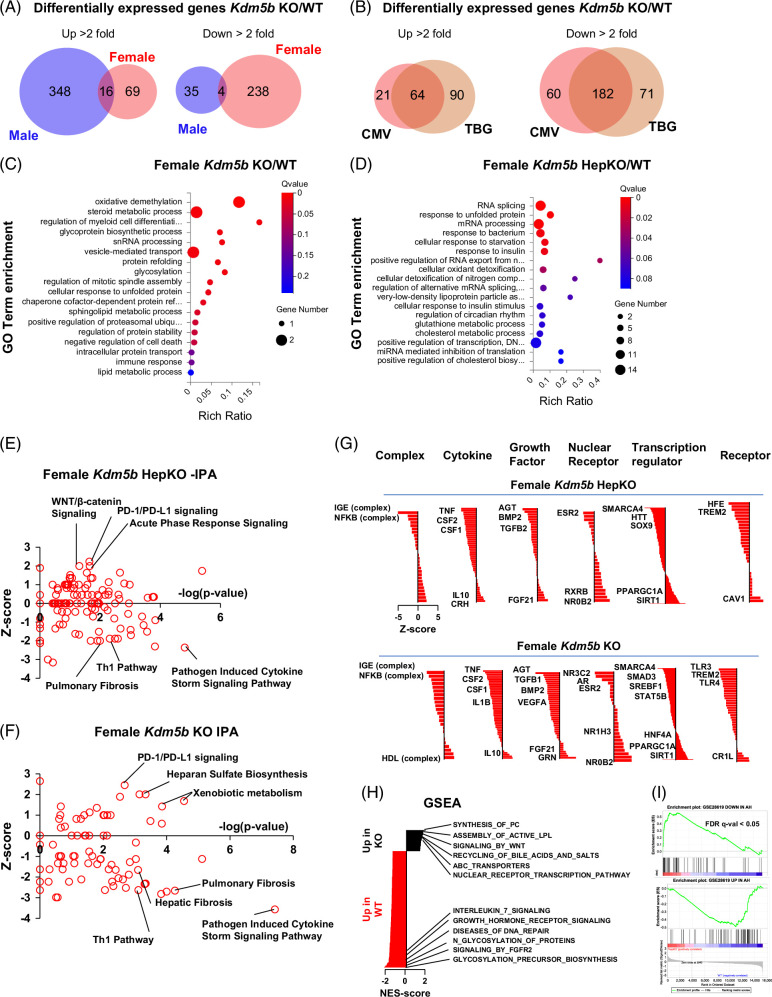
Hepatocyte KDM5B promotes alcohol-induced liver fibrosis and inflammation-related transcriptional changes in the liver. WT and *Kdm5b* KO mouse livers were analyzed by whole liver RNA sequencing. The RNA-seq data is available under the GSE number GSE244240. (A) Common and unique differentially expressed genes between male and female KO mice (CMV-Cre) compared to WT controls. (B) Common and unique differentially expressed genes between female KO mice (CMV-Cre) and female HepKO (TBG-Cre) compared to WT controls. (C, D) GO term enrichment analysis of differentially expressed genes in female *Kdm5b* KO mice (CMV-Cre) and female *Kdm5b* HepKO (TBG-Cre) compared to WT controls. (E, F) Ingenuity pathway analysis of differentially expressed genes in female KO mice (CMV-Cre) and female HepKO (TBG-Cre) compared to WT controls. (G) Ingenuity pathway analysis of upstream predicted regulators genes in female KO mice (CMV-Cre) and female HepKO (TBG-Cre) compared to WT controls, sorted by *Z*-score. (H) Gene set enrichment analysis of KEGG pathways regulated by hepatocyte-specific KDM5B KO in the liver. (I) Gene set enrichment analysis of AH-induced transcriptional changes (GSE28619) compared to KDM5B-dependent transcriptional changes. Abbreviations: IPA, Ingenuity pathway analysis; KO, knockout; WT, wild type.

To further assess the mechanism of KDM5B-mediated ALD development in females, we performed mRNA sequencing on whole liver from WT and KO mice fed 8 weeks of alcohol (Figure 4). We compared transcriptional changes in male and female *Kdm5b* KO mice (CMV-Cre) compared to WT controls (Figure 4A). As previously reported for shRNA-mediated knockdown studies, the effect of KO is sex specific. We found <3% of differentially regulated genes to be common between males and females (Figure 4A). When we compared CMV-Cre-mediated KO with hepatocyte-specific KO-induced changes, we found greater overlap, about 50% of differentially regulated genes were common between the 2 KO groups when compared to WT controls (Figure 4B).

GO term enrichment analysis highlighted the difference between CMV-Cre KO and hepatocyte-specific KO mice (Figures 4C, D). Notably, CMV-Cre-mediated KO affected genes related to protein glycosylation and vesicle-mediated transport, which were not affected by hepatocyte-specific KO. Both KO types affected genes involved in mRNA processing, immune response, and cholesterol metabolism.

Next, we examined activation and inhibition of specific pathways in WT and KO mice using the Ingenuity pathway analysis (IPA) tool (Figures 4E, F). IPA predicted that both KO groups showed suppression of pathways related to immune response and fibrosis, confirming that *Kdm5b* KO in hepatocytes reduced inflammation and fibrosis development in females. Further analysis of predicted upstream regulators in KO mice suggested that reduced immune cell activation (TNFα–NF-kB complex) and reduced stellate cell activation (TGFβ1–SMAD3) contribute to KO-mediated protection against alcohol-induced liver disease (Figures 4G, H). In addition, we observed that KO mice have a gene expression pattern that predicts an upstream regulator activation state consistent with factors that induce hepatocyte differentiation (more HNF4α, less SOX9) and an altered macrophage differentiation program (less CSF1 and TREM2).

We next examined pathways affected by *Kdm5b* KO using the Gene Set Enrichment Analysis (GSEA) tool and found that hepatocyte-specific *Kdm5b* KO induced activation of several metabolic pathways, such as ABC transporters, nuclear receptor–mediated transcription, LPL (lipoprotein lipase) secretion, and bile acid metabolism, suggesting that KO promoted liver metabolic functions (Figure 4H). In addition, we assessed the KO-induced changes relative to transcriptomic changes induced in human alcohol-associated hepatitis (AH) reported previously^36^ (Figure 4I). We found that genes downregulated in human AH were significantly upregulated in *Kdm5b* HepKO mice, while genes upregulated in AH were downregulated in *Kdm5b* KO mice.

Taken together, our data suggest that KDM5B KO prevents liver fibrosis development, preserves liver metabolic function in the presence of alcohol, and prevents AH-like transcriptional changes in the liver.

### KDM5B loss prevents alcohol-induced macrophage infiltration and hepatocytes dedifferentiation

**FIGURE 5:**
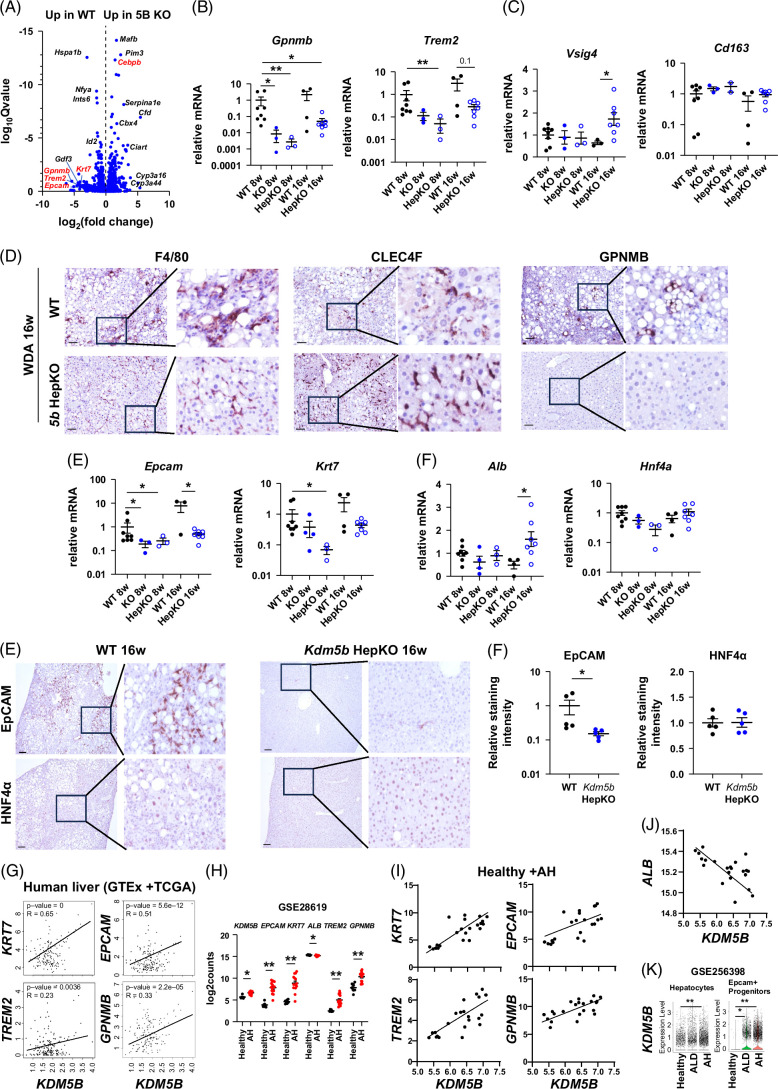
Hepatocyte KDM5B promotes alcohol-induced monocyte infiltration and ductular reaction. (A) Volcano plots of differentially expressed genes in female *Kdm5b* HepKO (TBG-Cre) compared to WT controls. (B, C) Relative whole liver mRNA in WT and *Kdm5b* KO mice after 8 or 16 weeks of WDA feeding. N=3–8 mice per group; *p<0.05 and **p<0.01. (D) Representative images of immunohistochemistry staining in WT and *Kdm5b* HepKO mice after 16 weeks of WDA feeding using antibodies specific for F4/80, CLEC4F, and GPNMB. (E) Representative images of EpCAM and HNF4α staining in WT and *Kdm5b* HepKO mice after 16 weeks of WDA feeding. (F) EpCAM and HNF4α positive staining intensity in these mice. N=5 mice per group; *p<0.05. (G) Human liver gene expression in GTEx and TCGA datasets (no tumor controls) combined. (H) Gene expression changes in human AH patients compared to healthy controls (GSE28619). (I, J) Correlation between KDM5B and indicated genes in healthy and AH samples. (K) snRNA-seq analysis of KDM5B gene expression in hepatocytes and EPCAM+ progenitors in healthy and ALD samples (GSE256398); **p<0.01. Scale bar 100 µm. Abbreviations: AH, alcohol-associated hepatitis; KO, knockout; WDA, western diet with alcohol; WT, wild type.

Among the top differentially regulated genes in KO mice, we found *Trem2* and *Gpnmb*, markers of lipid-associated macrophages derived from infiltrating monocytes (Figure 5A). We confirmed that both genes were significantly downregulated in KO mice at 8 weeks and 16 weeks of alcohol feeding, suggesting that monocyte infiltration is decreased in KO mice, in agreement with reduced chemokine gene expression (*Ccl2*, *Ccl5*) and other infiltrating monocyte markers (*Ccr2*, *Cx3cr1*). *Vsig4* and *Cd163* are the markers of mature KCs. These markers are lost or reduced during ALD progression, due to the loss of the mature KC phenotype. We found that although *Vsig4* levels were not different between WT and KO mice at 8 weeks of feeding, at 16 weeks of feeding, *Vsig4* levels were significantly upregulated in KO mice compared to WT controls, suggesting that *Kdm5b* KO prevents alcohol-induced loss of KC mature phenotype. CLEC4F is another well-established marker of mature KCs. We next assessed CLEC4F expression using immunohistochemistry together with F4/80 staining for total macrophages and GPNMB staining for lipid-associated macrophages (Figure 5D). We found that after 16 weeks of feeding F4/80 staining intensity was similar between WT and KO; however, at 8 weeks of feeding, the staining pattern was different. We found a reduced number of crown-like structures in KO mice. In contrast to F4/80 staining, the number of CLEC4F-positive cells was low in WT mice, suggesting that the majority of F4/80-positive macrophages were CLEC4F negative, suggesting the loss of the mature KC phenotype. While in KO mice, CLEC4F staining was greatly elevated compared to WT controls. In contrast, GPNMB staining, which was prominent in crown-like structures in WT mice, was absent in KO mice (Figure 5D).

Mature KCs are important for maintaining hepatocyte differentiation and preserving liver function in ALD.^37^ We next examined the markers of hepatocyte differentiation and hepatocyte progenitor markers in KO mice. We found that among the top differentially regulated genes, *Krt7* and *Epcam* were significantly downregulated in KO mice (Figures 5A, E) at both 8 weeks and at 16 weeks of alcohol feeding. These data correlated with reduced *Trem2* and *Gpnmb* expression. When we examined markers of hepatocyte differentiation (*Hnf4a* and *Alb*), we found that at 16 weeks of feeding, *Alb* levels were significantly upregulated in KO mice compared to WT controls, suggesting that *Kdm5b* KO prevents alcohol-induced loss of hepatocyte synthetic function (Figure 5F). We confirmed that EpCAM was significantly downregulated in KO mice compared to WT controls at 16 weeks of feeding by immunohistochemistry staining (Figures 5E, F). Moreover, in human liver samples from the GTEx and TCGA databases, we found a strong positive correlation between *KDM5B* and *KRT7/EPCAM* gene expression as well as a significant correlation between *KDM5B* and markers of infiltrating monocyte-derived macrophages (*TREM2*, *GPNMB*), suggesting that this mechanism exists in human liver as well.

We further examined this correlation in ALD patient samples. We found that in whole liver mRNA from AH patients (GSE28619), KDM5B was upregulated and correlated with *KRT7*, *EPCAM*, *TREM2*, and *GPNMB* gene expression (Figures 5H, I). In contrast, KDM5B strongly negatively correlated with *ALB* in these samples (Figure 5J). To evaluate cell-type-specific changes in KDM5B gene expression, we examined KDM5B expression in the snRNA-seq dataset (GSE 256389). We found that KDM5B was induced in hepatocytes and EPCAM+ progenitor-like cells from ALD patients, which correlated with loss of hepatocyte function and elevated progenitor gene expression in these patients (Figure 5K).

Taken together, these data suggest that *Kdm5b* KO in hepatocytes prevents alcohol-induced loss of KC and hepatocyte differentiation induced by alcohol.

### KDM5B loss in hepatocytes promotes macrophage C/EBPβ expression

**FIGURE 6:**
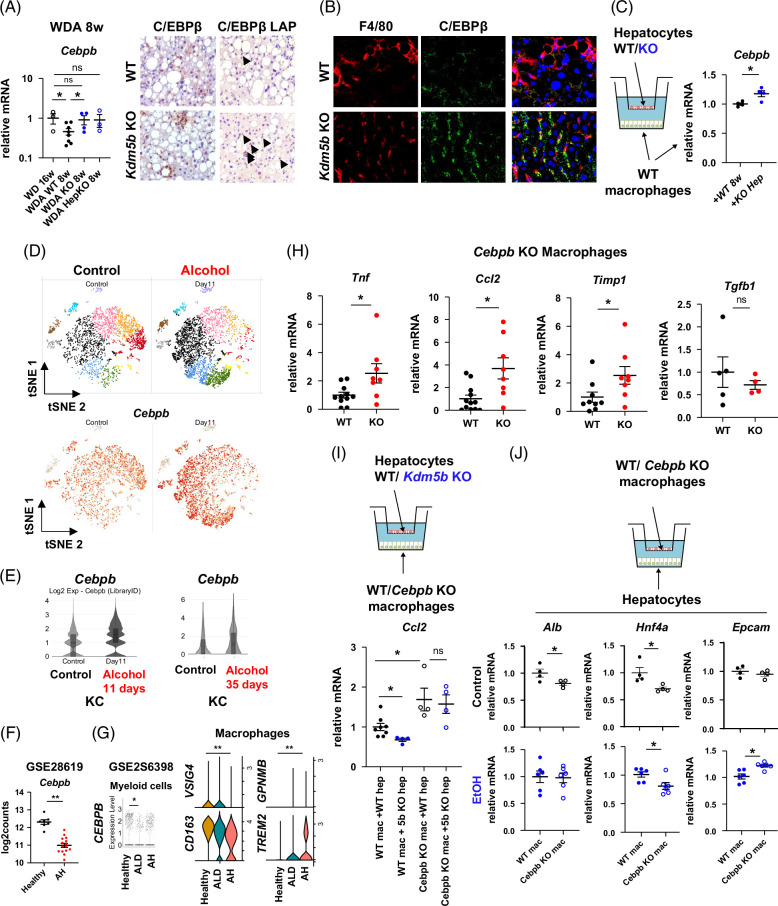
Hepatocyte KDM5B suppresses macrophage C/EBPβ expression in the liver. (A) Relative gene expression in whole liver samples from mice fed WD control or WDA for 8 weeks. (Right) Representative images of C/EBPβ staining in WT and *Kdm5b* KO mice after 8 weeks of WDA feeding. (B) Co-staining using F4/80-specific and C/EBPβ-specific antibodies in WT and *Kdm5b* KO mice after 8 weeks of WDA feeding. (C) *Kdm5b* WT or KO hepatocytes were used in a co-culture system with liver macrophages. Relative gene expression in macrophages after 24 hours of co-culture. N=3–6 independent experiments; *p<0.05. (D) The *Cebpb* gene is the top upregulated gene after 10 days of alcohol exposure in KCs. Mice were fed Lieber–DeCarli liquid diet or control for 11 days. FACS-sorted KCs were analyzed by single-cell RNA sequencing. *Cebpb* expression in these cells is indicated. (E) (Left) Violin plot of *Cebpb* gene expression in control-fed or alcohol-fed mice liver macrophages after 11 days of alcohol feeding. (Right) Violin plot of *Cebpb* gene expression in control-fed or alcohol-fed mice liver macrophages after 35 days of alcohol feeding. (F) Gene expression changes in human AH patients compared to healthy controls (GSE28619). (G) snRNA-seq analysis of gene expression in myeloid cells and macrophages in healthy and ALD samples (GSE256398); **p<0.01. (H) Peritoneal macrophages were isolated from WT (*Cebpb* *^fl/fl^*) or *Cebpb* MyeKO (*Cebpb* *^fl/fl^* Lyz2-Cre) mice. Relative gene expression in these macrophages. N=4–8 mice per group; *p<0.05. (I) Liver macrophages were isolated from WT (*Cebpb* *^fl/fl^*) or *Cebpb* MyeKO (*Cebpb* *^fl/fl^* Lyz2-Cre) mice and used for a co-culture study with WT or *Kdm5b* KO hepatocytes. Relative gene expression in macrophages. N=4 mice per group; *p<0.05. (J) Liver macrophages were isolated from WT (*Cebpb* *^fl/fl^*) or *Cebpb* MyeKO (*Cebpb* *^fl/fl^* Lyz2-Cre) mice and used for a co-culture study with WT hepatocytes in the presence or absence of alcohol. Relative gene expression in hepatocytes after 24 hours of co-culture. N=4–8 mice per group. Scale bar 100 µm. Abbreviations: ALD, alcohol-associated liver disease; AH, alcohol-associated hepatitis; KO, knockout; WD, western diet; WDA, western diet with alcohol; WT, wild type.

One of the top differentially regulated genes in *Kdm5b* KO mice was *Cebpb* (Figure 5A). We found that *Cebpb* level is decreased in alcohol-fed mice compared to control (WD only); however, the decrease is prevented by *Kdm5b* KO (Figure 6A). Specifically, C/EBPβ total protein as well as LAP isoform expression in non-parenchymal cells was increased in KO mice compared to WT controls (Figure 6A, right). Co-staining with the macrophage marker F4/80 suggested that C/EBPβ in macrophages was increased in *Kdm5b* KO mice compared to control (Figure 6B). Moreover, using a co-culture system, we found that compared to WT control hepatocytes, *Kdm5b* KO hepatocytes increased *Cebpb* gene expression in macrophages (Figure 6C), suggesting that C/EBPβ upregulation in *Kdm5b* KO mice could be a direct effect of the signals coming from *Kdm5b* KO hepatocytes.

To assess the role of C/EBPβ in liver macrophages after alcohol feeding, we examined *Cebpb* expression in KCs after 10 days of alcohol feeding. We found that *Cebpb* was the top gene upregulated in KC after 10 days of liquid diet alcohol feeding (Figure 6D, E), suggesting that C/EBPβ upregulation could be an early adaptation to alcohol. In agreement with that, *Cebpb* induction was lost after 5 weeks of alcohol feeding (Figure 6E).

Using human expression data, we found that *CEBPB* was downregulated in AH (Figure 6F) and specifically reduced in the myeloid cell cluster (Figure 6G). In addition, the *CEBPB* gene expression decrease correlated with altered macrophage identity marker gene expression. We found that ALD patient macrophages had reduced *CD163*, *VSIG4*, and increased *TREM2* and *GPNMB*, indicating that *CEBPB* downregulation correlates with the loss of the mature KC phenotype and an increase in the lipid-associated macrophage phenotype.

Next, we examined the phenotype of *Cebpb* KO macrophages compared to WT controls using peritoneal macrophages isolated from WT or *Cebpb* MyeKO mice. We found that *Cebpb* KO macrophages expressed higher levels of pro-inflammatory (*Tnf*, *Ccl2*) and pro-fibrotic genes (*Timp1*), suggesting that KDM5B-mediated macrophage C/EBPβ loss can contribute to alcohol-induced inflammation and fibrosis (Figure 6H). Finally, we confirmed that *Cebpb* KO in macrophages prevents the reduction in macrophage pro-inflammatory signaling induced by hepatocyte *Kdm5b* KO (Figure 6I), suggesting that the hepatocyte *Kdm5b* KO anti-inflammatory effect is in part due to upregulation of C/EBPβ in macrophages. Moreover, *Cebpb* KO in macrophages reduced hepatocyte differentiation markers (*Hnf4a*, *Alb*) and increased *Epcam* gene expression (Figure 6J), suggesting a bidirectional cell signaling process where hepatocyte *Kdm5b* expression regulates macrophage inflammatory phenotype through C/EBPβ, and macrophage C/EBPβ controls hepatocyte differentiation and function.

### Myeloid C/EBPβ controls KC identity and hepatocyte differentiation

**FIGURE 7:**
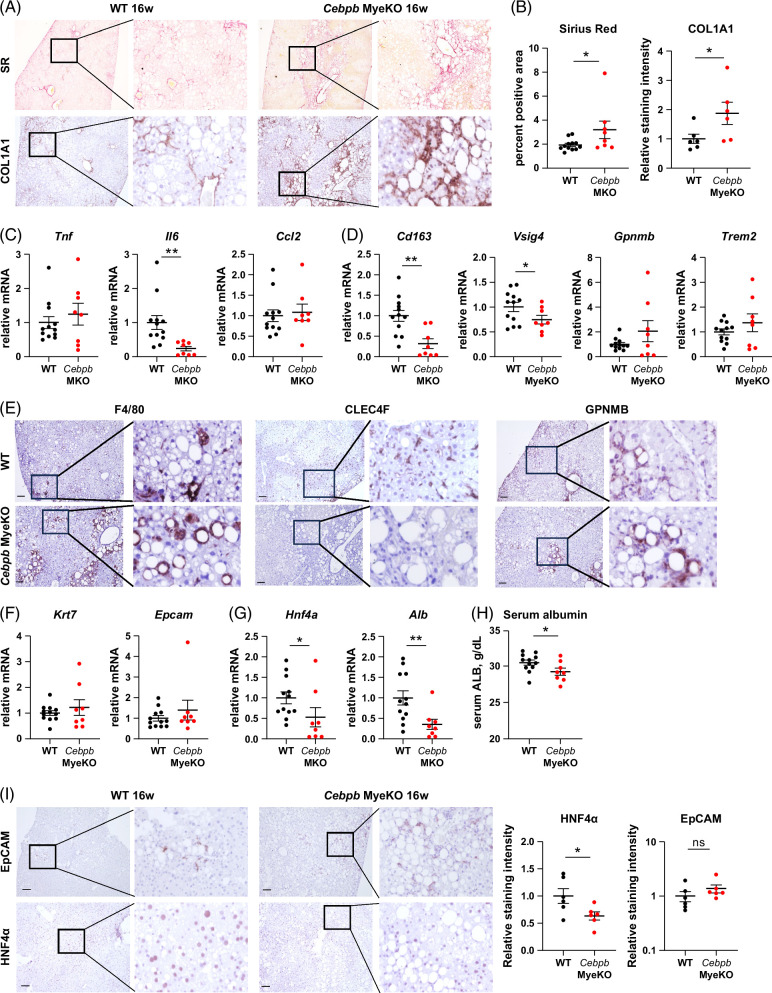
Myeloid C/EBPβ protects from hepatocyte dedifferentiation and loss of liver function in alcohol-fed mice. WT (*Cebpb* *^fl/fl^*) or *Cebpb* MyeKO (*Cebpb* *^fl/fl^* Lyz2-Cre) mice were fed WDA for 16 weeks. (A) Sirius Red and COL1A1 staining in these mice. (B) Sirius Red positive area in these mice. N=8–13 mice per group; *p<0.05. COL1A1-positive staining intensity in these mice. N=6 mice per group; *p<0.05. (C, D) Relative gene expression in whole liver mRNA in these mice. N=8–13 mice per group; *p<0.05 and **p<0.01. (E) Representative images of immunohistochemistry staining in WT and *Cebpb* MyeKO mice after 16 weeks of WDA feeding using antibodies specific for F4/80, CLEC4F, and GPNMB. (F, G) Relative gene expression in whole liver mRNA in these mice. N=8–13 mice per group; *p<0.05 and **p<0.01. (H) Serum albumin in these mice. N=8–13 mice per group; *p<0.05. (I) Representative images of EpCAM and HNF4α staining in these mice after 16 weeks of WDA feeding. (Right) EpCAM and HNF4α-positive staining intensity in these mice. N=6 mice per group; *p<0.05. Scale bar 100 µm. Abbreviations: KO, knockout; WDA, western diet with alcohol; WT, wild type.

To test the role of myeloid C/EBPβ in ALD development, we fed WT and *Cebpb* MyeKO mice a western diet and 20% alcohol (WDA) for 16 weeks. We found that *Cebpb* MyeKO mice showed an increase in Sirius Red and Collagen 1A1 staining (Figures 7A, B). Interestingly, WT and *Cebpb* MyeKO mice had similar levels of pro-inflammatory (*Tnf, Ccl2*) gene expression in whole liver mRNA, but showed lower expression of *Il6* (Figure 7C).

Next, we assessed markers of mature KCs and infiltrating monocyte-derived macrophages (Figure 7D). We found that *Cebpb* MyeKO mice showed a dramatic reduction in mature KC markers (*Cd163* and *Vsig4*), suggesting that C/EBPβ is required for maintaining the mature KC phenotype in alcohol-fed mice. In contrast, *Trem2* and *Gpnmb* expression was not altered in *Cebpb* MyeKO mice (Figure 7D). Next, we examined CLECF4 and GPNMB levels by immunohistochemistry (Figure 7E). We found that after 16 weeks of feeding, F4/80 and GPNMB levels were comparable; in contrast, CLEC4F staining was greatly decreased in *Cebpb* MyeKO mice compared to WT controls, suggesting that C/EBPβ is required for the mature KC phenotype in ALD (Figure 7E).

Since CD163-positive KCs are important for hepatocyte differentiation and maintenance of liver synthetic function in ALD,^37^ we assessed the markers of hepatocyte differentiation and progenitor marker expression (Figures 7F, G). We found that *Krt7* and *Epcam* were not altered in *Cebpb* MyeKO mice. In contrast, *Hnf4a* and *Alb* were significantly downregulated (Figure 7G), which correlated with decreased serum albumin levels in these mice (Figure 7H). We further confirmed significantly decreased HNF4α protein levels in the livers of *Cebpb* MyeKO mice by immunohistochemistry staining (Figure 7I).

**FIGURE 8:**
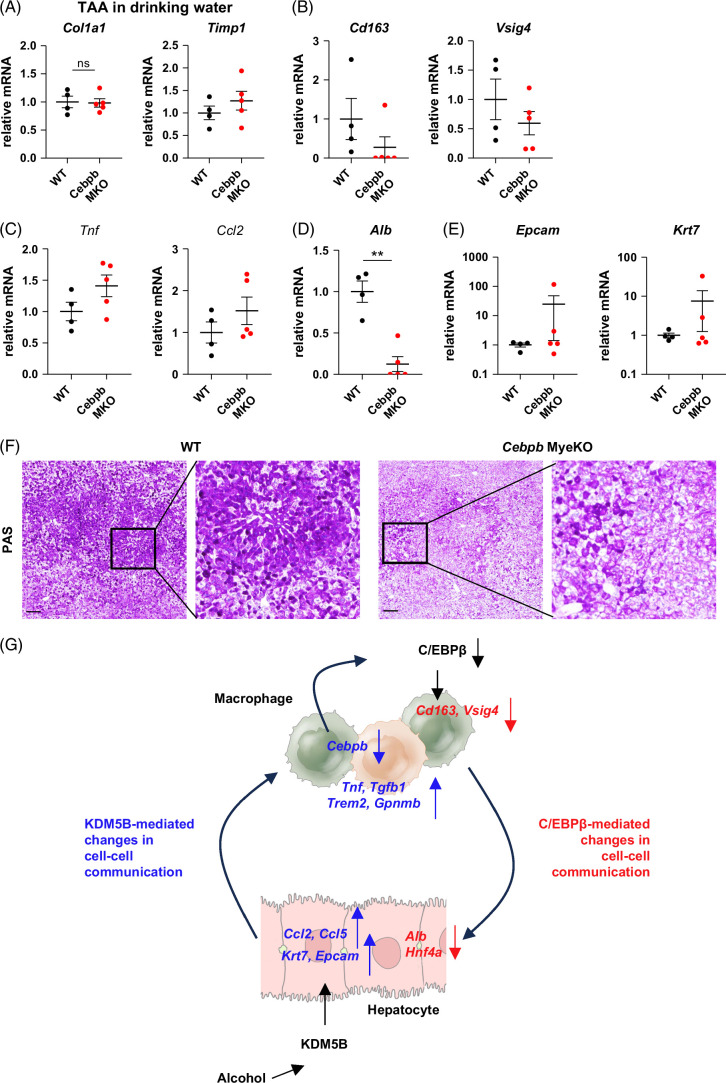
Myeloid C/EBPβ protects from loss of liver function in TAA-injured mice. WT (*Cebpb* *^fl/fl^*) or *Cebpb* MyeKO (*Cebpb* *^fl/fl^* Lyz2-Cre) mice were given TAA in the drinking water for 10 weeks. (A–E) Relative gene expression in whole liver mRNA in these mice. N=4–5 mice per group; **p<0.01. (F) Representative images of PAS staining in WT (*Cebpb* *^fl/fl^*) or *Cebpb* MyeKO (*Cebpb* *^fl/fl^* Lyz2-Cre) mice under normal conditions. (G) Model of alcohol-induced liver disease development mediated by KDM5B-dependent and C/EBPβ-dependent changes in cell–cell communication. Scale bar 100 µm. Abbreviations: PAS, periodic acid–Schiff; TAA, thioacetamide; WT, wild type.

To confirm that myeloid C/EBPβ expression is necessary for KC identity and hepatocyte differentiation, we tested WT and *Cebpb* MyeKO mice in the TAA-induced liver fibrosis model. After 10 weeks of TAA feeding, both groups of mice developed a similar degree of liver fibrosis (Figure 8A). However, *Cebpb* MyeKO mice showed a decrease in markers of mature KCs (Figure 8B), increased inflammatory gene expression (Figure 8C), and a dramatic decrease in *Alb* levels (Figure 8D) without a change in hepatocyte progenitor markers (Figure 8E). Finally, we examined the role of myeloid C/EBPβ in hepatocyte synthetic function by assessing glycogen levels in the livers of untreated WT and KO mice. We found that *Cebpb* MyeKO mice showed a decrease in glycogen levels under normal conditions, suggesting that C/EBPβ is necessary for glycogen storage as well (Figure 8F).

Taken together, these data suggest that alcohol-induced KDM5B-dependent C/EBPβ downregulation in myeloid cells suppresses KC identity and hepatocyte differentiation, and this creates a pathogenic cell–cell communication negative feedback loop between hepatocytes and macrophages that drives inflammation and hepatocyte dedifferentiation, which exacerbates ALD development (Figure 8G).

## DISCUSSION

ALD is the main cause of alcohol-associated mortality.^38^,^39^,^40^ Despite decades of studying molecular mechanisms involved in disease progression, therapeutic approaches are still limited.^38^,^40^,^41^ Recent advances in single-cell sequencing technology have highlighted the critical role of cell–cell interactions in the pathogenesis and complexity of the disease.^42^,^43^,^44^ Our recent studies suggest that pathological changes in hepatic cell–cell communication are mediated by alcohol-induced epigenetic changes in hepatocytes that alter hepatocyte-to-NPC signaling.^5^,^45^,^46^,^47^

We previously identified KDM5 histone demethylases as critical regulators of ALD development.^5^,^48^ In this work, we found that in females, alcohol promotes KDM5B-dependent changes in hepatocytes that reprogram stellate cells, liver macrophages, and endothelial cells to induce pro-inflammatory and pro-fibrotic signaling. Female hepatocyte-specific *Kdm5b* KO mice were protected from alcohol-induced NPC changes and did not develop alcohol-induced fibrosis and inflammation.

Previously, we identified that KDM5B in HSCs contributed to alcohol-induced fibrosis development in a sex-specific way.^5^,^35^ In this work, we found that hepatocyte KDM5B in females contributes significantly to alcohol-induced fibrosis development. Previously, we found that hepatocyte-specific KDM5B KO male mice were also partially protected from fibrosis development,^35^ suggesting that sex differences are largely mediated by KDM5B in non-parenchymal cells such as stellate cells.

However, sex differences in the effect of KDM5B-dependent changes in hepatocytes are also evident. Previously, we demonstrated that in males, KDM5B suppressed HNF4α expression and activity and albumin gene expression via direct binding to the gene promoters.^48^ Here we found that KDM5B also suppressed albumin expression in female mice. However, this was likely secondary to C/EBPβ downregulation in macrophages.

On the other hand, KDM5B promoted DR in female mice, which was absent in male mice (RNA-seq data). The extent of DR expansion in ALD correlates with disease progression and is associated with liver fibrosis and damage.^49^ These data suggest that KDM5B inhibition could be a potential target to modulate DR induced by alcohol.

We found that KDM5B in hepatocytes suppressed C/EBPβ gene and protein expression in liver macrophages. The role of C/EBPβ in KCs has not previously been well studied. Several in vitro studies suggested that C/EBPβ may promote pro-inflammatory gene expression. Other studies indicate the importance of C/EBPβ in anti-inflammatory (M2-like) macrophage changes. We found that macrophages isolated from C/EBPβ MyeKO mice had elevated pro-inflammatory gene expression, suggesting that C/EBPβ is likely anti-inflammatory in vivo. Interestingly, *Cebpb* was the top gene upregulated in KCs after only 10 days of alcohol exposure, suggesting that this induction is an early adaptation mechanism to prevent excessive liver inflammation due to alcohol-induced liver injury. Later loss of *Cebpb* induction at 5 weeks of alcohol exposure correlates with increased inflammation in these mice.^5^

Moreover, we found that macrophage C/EBPβ was essential for the expression of markers of mature KCs such as CLEC4F, indicating that C/EBPβ is essential for KC identity in ALD. In previous work, we identified that KDM5B in hepatocytes may suppress macrophage CD163 gene expression via loss of macrophage LXRα activity.^10^ Here we identified C/EBPβ-mediated *Cd163* gene expression as another mechanism of KC identity gene regulation, similarly dependent on KDM5B. KCs are essential in ALD livers for maintaining hepatocyte differentiation and preserving liver synthetic function. Loss of mature CLEC4F-positive KCs leads to a liver failure-like phenotype in alcohol-fed mice.^37^ Likewise, we found that loss of C/EBPβ in myeloid cells had similar effects. *Cebpb* MyeKO mice had dramatically reduced *Hnf4a* and *Alb* levels in the liver and reduced serum albumin level, highlighting the role of myeloid C/EBPβ in maintaining liver function. These data are in striking contrast to the role of C/EBPβ in hepatocytes, where C/EBPβ suppresses hepatocyte differentiation and promotes liver failure.^50^

Overall, we found that alcohol-induced changes in hepatocyte KDM5B activity promote hepatocyte chemokine expression (*Ccl2, Ccl5*), DR (EpCAM), and induce monocyte-derived macrophage infiltration (*Trem2, Gpnmb*) and loss of macrophage C/EBPβ. C/EBPβ loss, in turn, results in a loss of KC identity and reduction in hepatocyte differentiation (HNF4α) and albumin production. This pathogenic cell–cell communication feedback loop promotes liver inflammation, fibrosis, and loss of liver function (Figure 8G).

## Data Availability

All presented data and materials are available upon request. The RNA-seq data is available under the GSE number GSE244240.https://www.ncbi.nlm.nih.gov/geo/query/acc.cgi?acc=GSE244240. Irina Tikhanovich designed the study; Michael Schonfeld, Kruti Nataraj, and Samson Mah performed experiments; Irina Tikhanovich, Kruti Nataraj, and Michael Schonfeld performed data analysis; Kruti Nataraj, Steven Weinman, and Irina Tikhanovich wrote the manuscript. This study was supported by grants AA027586, AA031270, and AA012863 from the National Institute on Alcoholism and Alcohol Abuse, AI178204 from the National Institute of Allergy and Infectious Diseases, and VA Merit Award I01BX004694. The authors have no conflicts to report.

## References

[1] Osna NA Donohue TM Jr Kharbanda KK. Alcoholic liver disease: Pathogenesis and current management. Alcohol Res. 2017;38:147–161. 28988570 10.35946/arcr.v38.2.01 PMC5513682

[2] Pang JX Ross E Borman MA Zimmer S Kaplan GG Heitman SJ. Risk factors for mortality in patients with alcoholic hepatitis and assessment of prognostic models: A population-based study. Can J Gastroenterol Hepatol. 2015;29:131–138. 25855876 10.1155/2015/814827 PMC4399372

[3] Stickel F Hampe J. Genetic determinants of alcoholic liver disease. Gut. 2012;61:150–159. 22110053 10.1136/gutjnl-2011-301239

[4] Kamath PS Kim WR Advanced Liver Disease Study Group. The model for end-stage liver disease (MELD). Hepatology. 2007;45:797–805. 17326206 10.1002/hep.21563

[5] Schonfeld M Averilla J Gunewardena S Weinman SA Tikhanovich I. Alcohol-associated fibrosis in females is mediated by female-specific activation of lysine demethylases KDM5B and KDM5C. Hepatol Commun. 2022;6:2042–2057. 35468265 10.1002/hep4.1967 PMC9315128

[6] Han M Xu W Cheng P Jin H Wang X. Histone demethylase lysine demethylase 5B in development and cancer. Oncotarget. 2017;8:8980–8991. 27974677 10.18632/oncotarget.13858 PMC5352456

[7] Huang D Xiao F Hao H Hua F Luo Z Huang Z. JARID1B promotes colorectal cancer proliferation and Wnt/beta-catenin signaling via decreasing CDX2 level. Cell Commun Signal. 2020;18:169. 33109187 10.1186/s12964-020-00660-4 PMC7590656

[8] Mocavini I Pippa S Licursi V Paci P Trisciuoglio D Mannironi C. JARID1B expression and its function in DNA damage repair are tightly regulated by miRNAs in breast cancer. Cancer Sci. 2019;110:1232–1243. 30588710 10.1111/cas.13925 PMC6447846

[9] Shen X Zhuang Z Zhang Y Chen Z Shen L Pu W. JARID1B modulates lung cancer cell proliferation and invasion by regulating p53 expression. Tumour Biol. 2015;36:7133–7142. 25877751 10.1007/s13277-015-3418-y

[10] Schonfeld M O’Neil M Weinman SA Tikhanovich I. Alcohol-induced epigenetic changes prevent fibrosis resolution after alcohol cessation in mice. Hepatology. 2024;80:119–135. 37943941 10.1097/HEP.0000000000000675 PMC11078890

[11] Wang B Tan Y Zhang Y Zhang S Duan X Jiang Y. Loss of KDM5B ameliorates pathological cardiac fibrosis and dysfunction by epigenetically enhancing ATF3 expression. Exp Mol Med. 2022;54:2175–2187. 36481938 10.1038/s12276-022-00904-y PMC9794816

[12] Zhao Z Su Z Liang P Liu D Yang S Wu Y. USP38 couples histone ubiquitination and methylation via KDM5B to resolve inflammation. Adv Sci (Weinh). 2021;8:e2101964. 34165906 10.1002/advs.202101964 PMC8224409

[13] Jakobsen JS Waage J Rapin N Bisgaard HC Larsen FS Porse BT. Temporal mapping of CEBPA and CEBPB binding during liver regeneration reveals dynamic occupancy and specific regulatory codes for homeostatic and cell cycle gene batteries. Genome Res. 2013;23:592–603. 23403033 10.1101/gr.146399.112 PMC3613577

[14] Wang H Peiris TH Mowery A Le Lay J Gao Y Greenbaum LE. CCAAT/enhancer binding protein-beta is a transcriptional regulator of peroxisome-proliferator-activated receptor-gamma coactivator-1alpha in the regenerating liver. Mol Endocrinol. 2008;22:1596–1605. 18467525 10.1210/me.2007-0388 PMC2453599

[15] Wang B Gao C Ponder KP. C/EBPbeta contributes to hepatocyte growth factor-induced replication of rodent hepatocytes. J Hepatol. 2005;43:294–302. 15922473 10.1016/j.jhep.2005.02.029

[16] Luedde T Duderstadt M Streetz KL Tacke F Kubicka S Manns MP. C/EBP beta isoforms LIP and LAP modulate progression of the cell cycle in the regenerating mouse liver. Hepatology. 2004;40:356–365. 15368440 10.1002/hep.20333

[17] He L Ronis MJ Badger TM. Ethanol induction of class I alcohol dehydrogenase expression in the rat occurs through alterations in CCAAT/enhancer binding proteins beta and gamma. J Biol Chem. 2002;277:43572–43577. 12213809 10.1074/jbc.M204535200

[18] Trautwein C Rakemann T Pietrangelo A Plumpe J Montosi G Manns MP. C/EBP-beta/LAP controls down-regulation of albumin gene transcription during liver regeneration. J Biol Chem. 1996;271:22262–22270. 8703043 10.1074/jbc.271.36.22262

[19] Gao Y Sun W Shang W Li Y Zhang D Wang T. Lnc-C/EBPbeta negatively regulates the suppressive function of myeloid-derived suppressor cells. Cancer Immunol Res. 2018;6:1352–1363. 30171135 10.1158/2326-6066.CIR-18-0108

[20] McPeak MB Youssef D Williams DA Pritchett CL Yao ZQ McCall CE. Frontline Science: Myeloid cell-specific deletion of Cebpb decreases sepsis-induced immunosuppression in mice. J Leukoc Biol. 2017;102:191–200. 28476751 10.1189/jlb.4HI1216-537R PMC5505744

[21] Simpson-Abelson MR Hernandez-Mir G Childs EE Cruz JA Poholek AC Chattopadhyay A. CCAAT/enhancer-binding protein beta promotes pathogenesis of EAE. Cytokine. 2017;92:24–32. 28088614 10.1016/j.cyto.2017.01.005 PMC5337143

[22] Zhang DE Hetherington CJ Meyers S Rhoades KL Larson CJ Chen HM. CCAAT enhancer-binding protein (C/EBP) and AML1 (CBF alpha2) synergistically activate the macrophage colony-stimulating factor receptor promoter. Mol Cell Biol. 1996;16:1231–1240. 8622667 10.1128/mcb.16.3.1231 PMC231105

[23] Ren Q Liu Z Wu L Yin G Xie X Kong W. C/EBPbeta: The structure, regulation, and its roles in inflammation-related diseases. Biomed Pharmacother. 2023;169:115938. 38000353 10.1016/j.biopha.2023.115938

[24] Tanaka T Narazaki M Kishimoto T. IL-6 in inflammation, immunity, and disease. Cold Spring Harb Perspect Biol. 2014;6:a016295. 25190079 10.1101/cshperspect.a016295 PMC4176007

[25] Cloutier A Guindi C Larivee P Dubois CM Amrani A McDonald PP. Inflammatory cytokine production by human neutrophils involves C/EBP transcription factors. J Immunol. 2009;182:563–571. 19109189 10.4049/jimmunol.182.1.563

[26] Trautwein C Caelles C van der Geer P Hunter T Karin M Chojkier M. Transactivation by NF-IL6/LAP is enhanced by phosphorylation of its activation domain. Nature. 1993;364:544–547. 8336793 10.1038/364544a0

[27] Na YR Jung D Yoon BR Lee WW Seok SH. Endogenous prostaglandin E2 potentiates anti-inflammatory phenotype of macrophage through the CREB-C/EBP-beta cascade. Eur J Immunol. 2015;45:2661–2671. 26118414 10.1002/eji.201545471

[28] Lamkin DM Srivastava S Bradshaw KP Betz JE Muy KB Wiese AM. C/EBPbeta regulates the M2 transcriptome in beta-adrenergic-stimulated macrophages. Brain Behav Immun. 2019;80:839–848. 31132458 10.1016/j.bbi.2019.05.034 PMC6660400

[29] Ruffell D Mourkioti F Gambardella A Kirstetter P Lopez RG Rosenthal N. A CREB-C/EBPbeta cascade induces M2 macrophage-specific gene expression and promotes muscle injury repair. Proc Natl Acad Sci U S A. 2009;106:17475–17480. 19805133 10.1073/pnas.0908641106 PMC2762675

[30] Schonfeld M Zhao J Komatz A Weinman SA Tikhanovich I. The polymorphism rs975484 in the protein arginine methyltransferase 1 gene modulates expression of immune checkpoint genes in hepatocellular carcinoma. J Biol Chem. 2020;295:7126–7137. 32245889 10.1074/jbc.RA120.013401 PMC7242710

[31] Schonfeld M O’Neil M Villar MT Artigues A Averilla J Gunewardena S. A Western diet with alcohol in drinking water recapitulates features of alcohol-associated liver disease in mice. Alcohol Clin Exp Res. 2021;45:1980–1993. 34523155 10.1111/acer.14700 PMC9006178

[32] Troutman TD Bennett H Sakai M Seidman JS Heinz S Glass CK. Purification of mouse hepatic non-parenchymal cells or nuclei for use in ChIP-seq and other next-generation sequencing approaches. STAR Protoc. 2021;2:100363. 33748781 10.1016/j.xpro.2021.100363 PMC7960533

[33] Davies JQ Gordon S. Isolation and culture of murine macrophages. Methods Mol Biol. 2005;290:91–103. 15361657 10.1385/1-59259-838-2:091

[34] Tikhanovich I Zhao J Olson J Adams A Taylor R Bridges B. Protein arginine methyltransferase 1 modulates innate immune responses through regulation of peroxisome proliferator-activated receptor gamma-dependent macrophage differentiation. J Biol Chem. 2017;292:6882–6894. 28330868 10.1074/jbc.M117.778761 PMC5409459

[35] Nataraj K Schonfeld M Rodriguez A Sharma M Weinman S Tikhanovich I. Androgen effects on alcohol-induced liver fibrosis are controlled by a Notch-dependent epigenetic switch. Cell Mol Gastroenterol Hepatol. 2025;19:101414. 39349250 10.1016/j.jcmgh.2024.101414 PMC11609386

[36] Argemi J Latasa MU Atkinson SR Blokhin IO Massey V Gue JP. Defective HNF4alpha-dependent gene expression as a driver of hepatocellular failure in alcoholic hepatitis. Nat Commun. 2019;10:3126. 31311938 10.1038/s41467-019-11004-3 PMC6635373

[37] Sasaki K Rooge S Gunewardena S Hintz JA Ghosh P Pulido Ruiz IA. Kupffer cell diversity maintains liver function in alcohol-associated liver disease. Hepatology. 2025;81:870–887. 38687563 10.1097/HEP.0000000000000918 PMC11616785

[38] Farooq MO Bataller R. Pathogenesis and management of alcoholic liver disease. Dig Dis. 2016;34:347–355. 27170388 10.1159/000444545 PMC4910523

[39] Gonzalez-Reimers E Santolaria-Fernandez F Martin-Gonzalez MC Fernandez-Rodriguez CM Quintero-Platt G. Alcoholism: A systemic proinflammatory condition. World J Gastroenterol. 2014;20:14660–14671. 25356029 10.3748/wjg.v20.i40.14660 PMC4209532

[40] Orman ES Odena G Bataller R. Alcoholic liver disease: Pathogenesis, management, and novel targets for therapy. J Gastroenterol Hepatol. 2013;28(suppl 1):77–84. 23855300 10.1111/jgh.12030 PMC4405238

[41] Gao B Bataller R. Alcoholic liver disease: Pathogenesis and new therapeutic targets. Gastroenterology. 2011;141:1572–1585. 21920463 10.1053/j.gastro.2011.09.002 PMC3214974

[42] Andrews TS Atif J Liu JC Perciani CT Ma XZ Thoeni C. Single-cell, single-nucleus, and spatial RNA sequencing of the human liver identifies cholangiocyte and mesenchymal heterogeneity. Hepatol Commun. 2022;6:821–840. 34792289 10.1002/hep4.1854 PMC8948611

[43] Zhang W Conway SJ Liu Y Snider P Chen H Gao H. Heterogeneity of hepatic stellate cells in fibrogenesis of the liver: Insights from single-cell transcriptomic analysis in liver injury. Cells. 2021;10:2129. 34440898 10.3390/cells10082129 PMC8391930

[44] Xiong X Kuang H Ansari S Liu T Gong J Wang S. Landscape of intercellular crosstalk in healthy and NASH liver revealed by single-cell secretome gene analysis. Mol Cell. 2019;75:644–660.e5. 31398325 10.1016/j.molcel.2019.07.028 PMC7262680

[45] Schonfeld M Villar MT Artigues A Weinman SA Tikhanovich I. Arginine methylation of hepatic hnRNPH suppresses complement activation and systemic inflammation in alcohol-fed mice. Hepatol Commun. 2021;5:812–829. 34027271 10.1002/hep4.1674 PMC8122385

[46] Zhao J O’Neil M Schonfeld M Komatz A Weinman SA Tikhanovich I. Hepatocellular protein arginine methyltransferase 1 suppresses alcohol-induced hepatocellular carcinoma formation by inhibition of inducible nitric oxide synthase. Hepatol Commun. 2020;4:790–808. 32490317 10.1002/hep4.1488 PMC7262284

[47] Zhao J Adams A Weinman SA Tikhanovich I. Hepatocyte PRMT1 protects from alcohol induced liver injury by modulating oxidative stress responses. Sci Rep. 2019;9:9111. 31235809 10.1038/s41598-019-45585-2 PMC6591482

[48] Schonfeld M Averilla J Gunewardena S Weinman SA Tikhanovich I. Male-specific activation of lysine demethylases 5B and 5C mediates alcohol-induced liver injury and hepatocyte dedifferentiation. Hepatol Commun. 2022;6:1373–1391. 35084807 10.1002/hep4.1895 PMC9134811

[49] Irvine KM Clouston AD Gadd VL Miller GC Wong WY Melino M. Deletion of Wntless in myeloid cells exacerbates liver fibrosis and the ductular reaction in chronic liver injury. Fibrogenesis Tissue Repair. 2015;8:19. 26473015 10.1186/s13069-015-0036-7 PMC4606475

[50] Elias G Schonfeld M Saleh S Parrish M Barmanova M Weinman SA. Sepsis-induced endothelial dysfunction drives acute-on-chronic liver failure through Angiopoietin-2–HGF-C/EBPbeta pathway. Hepatology. 2023;78:803–819. 36943063 10.1097/HEP.0000000000000354 PMC10440279

